# Multi-center MRI prediction models: Predicting sex and illness course in first episode psychosis patients

**DOI:** 10.1016/j.neuroimage.2016.07.027

**Published:** 2017-01-15

**Authors:** Mireille Nieuwenhuis, Hugo G. Schnack, Neeltje E. van Haren, Julia Lappin, Craig Morgan, Antje A. Reinders, Diana Gutierrez-Tordesillas, Roberto Roiz-Santiañez, Maristela S. Schaufelberger, Pedro G. Rosa, Marcus V. Zanetti, Geraldo F. Busatto, Benedicto Crespo-Facorro, Patrick D. McGorry, Dennis Velakoulis, Christos Pantelis, Stephen J. Wood, René S. Kahn, Janaina Mourao-Miranda, Paola Dazzan

**Affiliations:** a Department of Psychiatry, Rudolf Magnus Institute of Neuroscience, University Medical Center Utrecht, Utrecht, The Netherlands; b Department of Psychosis Studies, Institute of Psychiatry, King's College London, UK; c Center for Epidemiology and Public Health, Health Service and Population Research Department, Institute of Psychiatry, King's College, London, UK; d Neuroimaging Unit, Technological Facilities, Valdecilla Biomedical Research Institute IDIVAL, Santander, Cantabria, Spain; e CIBERSAM, Centro Investigación Biomédica en Red de Salud Mental, Department of Psychiatry, School of Medicine, University of Cantabria, Santander, Spain; f Laboratory of Psychiatric Neuroimaging, Department and Institute of Psychiatry, Faculty of Medicine, University of São Paulo, Centro de Medicina Nuclear, São Paulo, SP, Brazil; g Department of Psychiatry, Melbourne Neuropsychiatry Center, University of Melbourne, Melbourne, Australia; h School of Psychology, University of Birmingham, UK; i Department of Computer Science, University College London, London, UK; j Max Planck University College London Centre for Computational Psychiatry and Ageing Research, University College London, London, UK; k NIHR Mental Health Biomedical Research Center at South London and Maudsley NHS Foundation Trust and King's College, London, UK; l Department of Medicine and Psychiatry, University Hospital Marques de Valdecilla, School of Medicine, University of Cantabria-IDIVAL-CIBERSAM, Santander, Spain

## Abstract

Structural Magnetic Resonance Imaging (MRI) studies have attempted to use brain measures obtained at the first-episode of psychosis to predict subsequent outcome, with inconsistent results. Thus, there is a real need to validate the utility of brain measures in the prediction of outcome using large datasets, from independent samples, obtained with different protocols and from different MRI scanners.

This study had three main aims: 1) to investigate whether structural MRI data from multiple centers can be combined to create a machine-learning model able to predict a strong biological variable like sex; 2) to replicate our previous finding that an MRI scan obtained at first episode significantly predicts subsequent illness course in other independent datasets; and finally, 3) to test whether these datasets can be combined to generate multicenter models with better accuracy in the prediction of illness course.

The multi-center sample included brain structural MRI scans from 256 males and 133 females patients with first episode psychosis, acquired in five centers: University Medical Center Utrecht (The Netherlands) (n = 67); Institute of Psychiatry, Psychology and Neuroscience, London (United Kingdom) (n = 97); University of São Paulo (Brazil) (n = 64); University of Cantabria, Santander (Spain) (n = 107); and University of Melbourne (Australia) (n = 54). All images were acquired on 1.5-Tesla scanners and all centers provided information on illness course during a follow-up period ranging 3 to 7 years. We only included in the analyses of outcome prediction patients for whom illness course was categorized as either “continuous” (n = 94) or “remitting” (n = 118).

Using structural brain scans from all centers, sex was predicted with significant accuracy (89%; p < 0.001). In the single- or multi-center models, illness course could not be predicted with significant accuracy. However, when reducing heterogeneity by restricting the analyses to male patients only, classification accuracy improved in some samples.

This study provides proof of concept that combining multi-center MRI data to create a well performing classification model is possible. However, to create complex multi-center models that perform accurately, each center should contribute a sample either large or homogeneous enough to first allow accurate classification within the single-center.

## Highlights

• Multi-center neuroimaging data can be combined to classify clear biological outcome.
• Classification is only significant in centers with similar illness outcome definition.
• Multi-center models can increase performance of smaller and heterogeneous samples.
• Multi-center studies should use large samples and standardized clinical information.
• Multi-center models have the potential to yield clinically useful predictions.

## 1 Introduction

Population-based studies indicate a lifetime prevalence of psychoses above 3% (Perälä and Suvisaari, 2007). While one third of affected individuals experience psychotic symptoms for a short period of time, others are affected throughout their entire lives (Harrison et al., 2001). Unfortunately, there is no way to predict, early on, which individuals will develop this incapacitating course. Identifying these individuals at illness onset is important as it could help provide better care to those most at risk.

Schizophrenia, and to a lesser extent other psychotic disorders, are associated with smaller gray matter volume, predominantly in the prefrontal cortex, but also in superior and medial frontal and temporal gyri, insula and thalamus (Fornito et al., 2009, Haijma et al., 2013, Honea et al., 2000, Shepherd et al., 2012). Even at the first psychotic episode, patients already show thalamic, insular and hippocampal volume reductions and larger ventricular volume compared to healthy controls (Levitt et al., 2010, Rosa et al., 2010, Schaufelberger et al., 2007, Steen et al., 2006).

Several structural Magnetic Resonance Imaging (MRI) studies have tried to use these brain measures at the time of the first episode to predict subsequent outcome. While many did not find significant differences between poor and good outcome patients (Molina et al., 2010, van Haren et al., 2003), others reported contradictory findings. A 1-year follow-up study found that first-episode schizophrenia (FE-SZ) patients with clinical deterioration had a smaller area of internal capsule compared to those with stable psychopathology, but no differences in either its volume or in any of the other 32 regions of interest (ROI) studied (Wobrock et al., 2009). Another short-term study found that volume of the dorsolateral prefrontal cortex was predictive of outcome at 1-year, but not at 2-years (Prasad et al., 2005). Studies with longer follow-up periods – 5 to 6 years – more consistently found that smaller initial gray matter volume was predictive of poorer outcome at follow-up (Cahn et al., 2006, Lieberman et al., 2001, Milev et al., 2003). This evidence suggests that evaluating outcome over longer follow-up periods could potentially improve the predictive power of brain measures.

The studies described above used a univariate approach to identify brain differences related to subsequent outcome. While this statistical approach allows for inferences about regional effects, it does not enable predictions at the level of the individual subject. More recently, machine-learning approaches have shown potential for clinical translation (Fu and Costafreda, 2013), and multivariate pattern recognition techniques have been applied to MRI data for the individualized prediction of clinical characteristics. Pattern recognition is a field within the area of machine learning concerned with automatic discovery of regularities in the data through the use of computer algorithms, and with using these regularities to classifying data into different categories (Bishop, 2006). When applied to data such as structural MRI, brain scans are treated as spatial patterns, and pattern recognition models are used to identify statistical properties of the data, which in turn enable discrimination between groups of subjects, for example patients from healthy subjects (Kambeitz et al., 2015, Klöppel et al., 2012, Nieuwenhuis et al., 2012, Orrù et al., 2012). This application has shown promising results. For example, even in first episode patients, multivariable models have been used to predict diagnoses with accuracies ranging between 79.3% and 91.5% (Karageorgiou et al., 2011, Pohl and Sabuncu, 2009, Sun et al., 2009, Takayanagi et al., 2011, Takayanagi et al., 2010). Furthermore, our own previous work has shown that MRIs obtained at time of the first psychotic episode (in a sample of 56 patients) could be used to predict illness course 6 years later with an accuracy of approximately 70% (Mourao-Miranda et al., 2012). This accuracy is substantially higher than that reported in an earlier one-year follow-up study that used pattern recognition to separate remitting (n = 15) and not remitting (n = 21) patients, which only achieved an accuracy of 58% (Zanetti et al., 2013). Although these results are modest, they support the potential clinical utility of biological brain markers for the prediction of outcome in schizophrenia.

One of the biggest challenges in the translation of neuroimaging findings into clinical practice is the need to validate these models across large independent samples and across data obtained from different MRI scanners (Schnack et al., 2010). This is essential to demonstrate robustness in the variability introduced by factors such as scanner type, acquisition protocols and clinical evaluation. In addition, combining multiple samples increases the overall sample size, overcoming a limitation common to many neuroimaging studies. In fact, recent studies on Alzheimer's disease (Dukart et al., 2013, Dyrba et al., 2013, Li et al., 2014) and major depression (Mwangi et al., 2012) have used multi-center data and shown very high classification accuracies, ranging from ~ 80% to ~ 90%.

In this study we combined five independent structural MRI datasets from leading international centers for the study of psychosis (Institute of Psychiatry, Psychology and Neuroscience, London; University Medical Center, Utrecht; University of São Paulo, São Paulo; University of Cantabria, Santander; University of Melbourne, Melbourne). We included both affective and non-affective psychoses for two reasons: first, because several brain alterations are common to both; and second, because the ability of the MRI obtained at first episode to predict subsequent outcome should be tested across all psychoses, since more specific diagnoses are still uncertain when the MRI is acquired. We had three main aims: 1) to investigate whether structural MRI data from multiple centers can be combined to create a machine-learning model able to predict a strong biological variable like sex; 2) to replicate our previous finding from the London dataset that an MRI scan obtained at first episode can be used to predict subsequent illness course in four independent datasets (Mourao-Miranda et al., 2012); and finally, 3) to test whether these datasets can be combined to generate multicenter models with better accuracy in the prediction of illness course.

We hypothesized that, by combining data from different scanners and thus creating larger samples, it would be easier for the classifier to learn the classification task and for any scanner or site effects to be considered as noise, thus resulting in a more robust model and in higher classification accuracy (of both sex and outcome). We therefore expected that the models would replicate our previous finding that an MRI at first episode can be used to predict subsequent illness course, with significant accuracy.

## 2 Method

### 2.1 Samples

The overall sample comprised five datasets of patients (total n = 389) who had an MRI scan at the time of their first episode of any psychotic illness (including DSM-IV diagnosis of schizophrenia, schizophreniform disorder, schizotypal disorder, schizoaffective disorder, depression with psychotic symptoms, bipolar affective disorder, psychosis not otherwise specified), and who were followed up over a period ranging between three and seven years, when clinical outcome was evaluated. The samples included: n = 67 patients from the University Medical Center Utrecht (UMCU, The Netherlands) (Cahn et al., 2002); n = 97 patients from the Institute of Psychiatry, Psychology and Neuroscience (IoPPN, London, United Kingdom) (Mourao-Miranda et al., 2012) from the initial study; n = 64 patients from the University of São Paulo (São Paulo, Brazil) (Schaufelberger et al., 2007); n = 107 patients from the University of Cantabria (Santander, Spain) (Crespo-Facorro et al., 2009); and n = 54 patients from the University of Melbourne (Melbourne, Australia) (Velakoulis et al., 2006). The samples were derived from well-established studies, the main findings of which have been extensively published elsewhere. All subjects were scanned in a 1.5 T scanner (protocol details provided below). All participants gave written informed consent and local ethics committees approved the studies.

### 2.2 Sex groups

Although classifying sex is a complex task, the classification problem is relatively easy, as sex can be unequivocally determined and brain sexual dimorphisms is well established, particularly in the Heschl's gyrus, the planum temporale and the hippocampal formation (Good et al., 2001). The dataset used in this analysis included 133 females and 256 males. To create a model based on a balanced dataset, all 133 female subjects were included, independently of their illness course, together with a subset of 133 males randomly selected from the overall sample, matched for site. To compare multi-center models to single-center models, five single-center models were created with all the females and a random selection of males. To reduce chances of selection bias, we built one hundred models with these random selections. Cross-validation (see details below) was used to estimate average prediction percentages.

### 2.3 Outcome groups

All centers provided information on the number of episodes that patients had experienced during the follow-up period and on whether they had achieved remission. Centers differed in the instruments used to evaluate illness course during follow-up (Table 2). These included: World Health Organization Life Chart (World Health organization, 1992), Schedules for Clinical Assessment in Neuropsychiatry (SCAN (Wing et al., 1990)), Positive and Negative Syndrome Scale, (PANNS (Kay et al., 1989)), Comprehensive Assessment of Symptoms and History, (CASH (Andreasen et al., 1992)) and Scale for the Assessment of Negative Symptoms (SANS, (Andreasen, 1984)). Illness course was therefore classified using a conservative approach into two groups that captured patient with an “extreme” type of outcome: one group with a “continuous” illness course (no remission of symptoms of > 6 months); and one group with a “remitting” illness course (one or more periods of remission of at least 6 months, and no episode lasting longer than 6 months). All patients who had experienced only a single psychotic episode (lasting no longer than 6 months) were included in the “remitting” group. Three centers (London, Utrecht, São Paulo) provided additional information on duration of the psychotic episodes. Patients who were neither in the continuous nor in the remitting group (i.e., had a remission and an episode lasting longer than 6 months) were excluded from further analyses on illness course (although they were included in the sex-based analyses). Within the London, Melbourne and Utrecht samples, 59%, 56% and 58% of all patients were included respectively. In the samples from Santander and São Paulo the percentages were 53% and 45% of the entire samples respectively. In total, 94 continuous patients and 118 remitting patients were included in the outcome analyses. Most patients had a diagnosis of schizophrenia (n = 114), followed by schizophreniform disorder and schizoaffective disorder (n = 39). Other diagnoses included bipolar affective disorder (n = 13), brief psychotic disorder (n = 5) and depression with psychotic symptoms (n = 9). The mean duration of follow-up in years was 6.3 (SD = 2.2), 4.9 (SD = 0.8), 7.0 (SD = 1.4), 3.0 (SD = 0.0) and 3.7 (SD = 0.6) respectively for London, Utrecht, Melbourne, Santander, and São Paulo.

After excluding patients who could not be classified as either continuous or remitting, the sample included 141 male patients (66 remitting and 75 continuous) and 71 females (52 remitting and only 19 continuous). To model less heterogeneous samples, we only included male patients, since the number of females per illness-course group per center was very small, and the samples size would have been too small to create a female-only model (see Table 1 for details).

### 2.4 MRI protocols and processing

All images were acquired on 1.5-Tesla scanners (see Table 3). The T1-weighted images were pre-processed according with the same protocol. Scans were manually oriented into MNI space, after which a non-uniformity correction was applied to remove radio frequency (RF) field inhomogeneity (Sled et al., 1998). Spatially normalized gray matter probabilities were obtained by running “segment” in SPM8 (Ashburner and Friston, 2005). This method segments, spatially normalizes (modulated normalized) and bias-corrects (10 full width half maximum (FWHM) 150 mm) all brains into the same space and dimensions (dimension: 91 × 109 × 91; voxel size: 2 × 2 × 2 mm). To reduce noise, all scans were smoothed with a 4-mm FWHM Gaussian kernel.

To ensure that only actual gray matter was included in the analysis, voxels with gray matter probabilities below 0.03 were excluded from the analysis. This resulted in 170,000 voxels or features per subject being included in the analyses.

### 2.5 Pattern recognition analyses

All models were created using a linear Support Vector Machine (SVM) (Vapnik, 1999), which is a supervised machine learning method commonly applied to binary classification problems in neuroimaging (Klöppel et al., 2012, Orrù et al., 2012). In supervised learning approaches, a predictive function is “learned” from labeled training data, which is a data set consisting of examples (e.g. gray matter patterns) and labels (e.g. patients or healthy controls). The binary problem in this case consists of classification of two previously defined groups, for example, female vs. male or remitting vs. continuous. Every subject is represented by its gray matter probability map, which defines a high dimensional feature vector (in which each voxel in the map corresponds to a feature in the feature-vector). In order to build the binary classifier, the labeled data are used to create a model or decision boundary based on training examples (such as gray matter probability maps from remitting and continuous course patients). In the linear case, this decision boundary corresponds to a hyperplane in the voxel space. The SVM finds the hyperplane that has the largest margin or separation between the two groups, also known as optimal hyperplane (Vapnik, 1999). The advantages of SVM compared to other classification techniques are its scalability and computational efficiency in higher dimensional problems.

A pre-existing implementation (Chang and Lin, 2009) of LIBSVM in Matlab (version 2009b) was used to compute models with a linear kernel. The parameter C was determined through nested cross validation. Nested cross validation involves one more loop than normal cross validation (explained below). The inner loop is used for optimizing model parameters and the outer loop is used to estimate model performance based on the test subjects, which are not used during the parameter optimization process.

Cross validation is a technique that estimates model performance using parts of the sample for training and testing. One part of the data is used for model estimation or training, and the other part is used for model testing. We elected to use a leave-two-out cross-validation framework (L2o), which allows for one subject of each class to be left out for testing, and for the remaining subjects from both classes to be used for model creation. In our multi-center models, the pairs that were left out were always from the same center. To obtain a more generalizable predictive value, we bootstrapped one hundred models: each time, a balanced group (as large as possible and with an equal number of subjects per class) was selected randomly, and then a completely independent leave-two-out cross-validation was performed. The average of all these bootstraps is what generated our predictive values.

### 2.6 Performance measures

In the sex classification model, the percentage of correctly classified females and males was estimated as the correctly classified females divided by all the females, and the correctly classified males divided by all the males.

The performance of the disease course models is reflected in a value per class: the positive and negative predictive accuracies (PPA and NPA). These are the ratios of correctly classified subjects in a specific class (true positives (TP) or true negatives (TN)), divided by all the subjects classified as belonging to that class (TP + false positives (FP) or TN + false negatives (FN)):

$$\text{Positive predictive accuracy}=\mathrm{TP}/\left(\mathrm{TP}+\mathrm{FP}\right)$$

$$\text{Negative predictive accuracy}=\mathrm{TN}/\left(\mathrm{TN}+\mathrm{FN}\right)$$

Significance of the models was determined by permutation testing. During the permutation test the labels were permuted one thousand times before re-training the models. The occurrence of accuracies equal to- or higher than- the accuracy of the model that is being tested were counted and then divided by the number of permutations, resulting in their p-value.

## 3 Results

### 3.1 Sex classification models

The five single-center model accuracies are presented in Table 4 (a). The centers with the larger samples, London and Santander, performed 5–10% better than the other, smaller sample centers. The left part of the table contains the results of the multi-center support-vector-machine (SVM)-model, in which data from all centers were combined to train the models. The average accuracy in sex classification was 89% (range 81% to 94%) (males = 88%, females = 90%, p = 0.001). This multi-center model performed as well as the single-center model of the two larger samples (London 89% and Santander 88% vs. multi-center 89%). Interestingly, in the multi-center model, the accuracies of the centers with smaller samples (Utrecht and Melbourne) improved considerably, especially in the percentage of correctly classified females. Overall, the accuracy improved by 2% to 9% when compared to the single-center models. Moreover, the difference in classification accuracy of males and females was much smaller, indicating that the model was as likely to correctly classify a male as it was to correctly classify a female. The large differences we had seen in the smaller single-center models (Utrecht (20%), Melbourne (9%) and São Paulo (10%)) were reduced to only 6%, 5% and 0.3% respectively in the combined model.

### 3.2 Illness course classification in individual centers

To investigate whether gray matter density at first episode could predict illness course, each dataset was first analyzed individually (Table 4, b). When both male and female patients were included, the classification into continuous and remitting course was significant and above chance only in the sample from London at 68% and 70% (p < 0.02 and p < 0.007 respectively).

To investigate if a less heterogeneous sample would lead to better models, we built new models including only male patients (Table 4, c). This increased the average accuracy in the Santander and São Paulo datasets by 11% and 15% respectively. However, it negatively affected the average classification accuracy in London (from 69% to 66%), while it did not significantly affect the accuracy in the datasets from Utrecht and Melbourne. Only the accuracy in the sample from São Paulo reached significance (p = 0.005), and the remitting patients from London were classified with a significance of p = 0.08. The lack of significance in the other samples may be due to the small sample sizes. The lack of improvement in accuracy after reducing data heterogeneity suggests that heterogeneity was not the only factor leading to the poor performance of these models. Reducing heterogeneity also led to smaller sample sizes, which could have negatively affected the results.

### 3.3 Illness course classification with multi-center models

In the third and last set of analyses we combined all data into multi-center models to classify illness course. Combining data from all centers into one model did not improve the results obtained from single-center models. The classification accuracy remained at chance level and did not reach significance (Table 4, b).

**Fig. 1:**
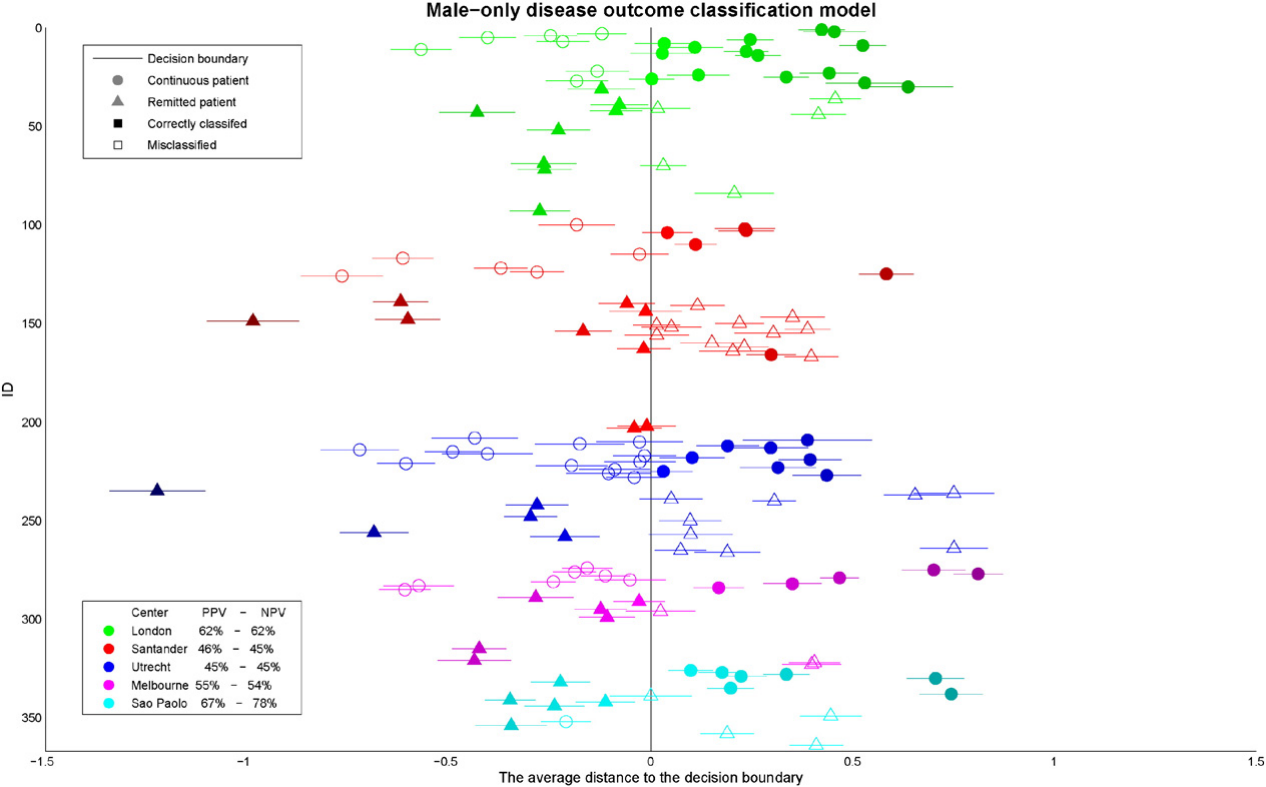
Depicts the results of multi-center male-only illness course-classification; the colors represent subjects from the different centers. The vertical black line represents the decision boundary. Ideally, all continuous patients (circles) would appear right of the line and the remitting patients (triangles) left of the line.

The less heterogeneous multi-center model, including only male patients, showed that illness course in subjects from London and São Paulo was accurately classified with an average of 62% and 74% respectively (depicted in green and light blue in Fig. 1). Unfortunately, combining all five centers did not increase the accuracies in the others centers.

## 4 Discussion

To the best of our knowledge, this is the first study that has investigated whether neuroimaging data obtained at the first psychotic episode from different scanners and with different protocols can be combined and used to accurately predict subsequent illness course. Our main finding is that combining multi-center neuroimaging data can lead to a single model that performs well (up to 90% accurate) when classifying strong, reliable biological outcomes such as sex. In contrast, our second main finding is that when classifying clinical characteristics, such as illness course, classification accuracies are only modest, and significant only in centers with most similar definitions of outcome. In addition, the results show that multi-center models can be used to increase the performance of smaller and heterogeneous samples. Taken together, these findings suggest that with larger samples, standardized clinical information and clear-cut outcome groups, multi-center-models have the potential to yield generalizable, clinically useful predictions.

### 4.1 Sex classification models

Can we pool data from different centers to increase classification accuracy and create better predictive models? The models based on relatively larger samples (London and Santander) correctly classified up to 90% of individuals from both sexes, while those based on smaller samples (Melbourne and São Paulo's) classified males with approximately 88% accuracy, and females with a lower accuracy (mean 79%). This could be due to the smaller number of females in the models: consistently with previous studies, models based on small samples show larger fluctuations in classification accuracies (Nieuwenhuis et al., 2012). We showed that by combining data from multiple centers (133 females and 133 males), the accuracy of the model improved (by approximately 8%), including in those centers with small samples (for example, the Utrecht sample only included 9 females). This confirms our hypothesis that combining datasets, even if acquired on different scanners and from different centers, can theoretically improve predictive models. The limitations of small sample sizes can therefore be overcome by combining multi-center-data, and unbalanced datasets (with fewer subjects of one group and more of the other group) can benefit from the more balanced distribution that derives from merging multiple datasets. However, it should be noted that our findings do not aim to provide information on brain sex dimorphism in psychosis, particularly in the absence of a non-psychosis comparison group, and only provide proof of concept for accurate multicenter classification models in the presence of a reliable outcome.

### 4.2 Illness course classification

Our second main finding was that when creating single-center models to predict future outcome, only one center (London) achieved a significant accuracy (Zanetti et al., 2013). Classifying illness course is much more challenging than classifying sex. The lower accuracies observed in Utrecht, Melbourne, Santander and São Paulo are probably due to the small sample size in these centers (ranging between 10 and 16 subjects). In addition, illness course is much more heterogeneous than what can be captured by a simple continuous vs. remitting classification, and clinical differences between these two course types could be extremely subtle and difficult to identify. This highlights the importance of using strong, valid and standardized instruments for the classification of outcome (Fu and Costafreda, 2013, Mayberg, 2014).

### 4.3 Multi-center illness course classification

The same modest results were seen when data from the five centers were combined to predict illness course. Although classifying outcome is more complex than classifying sex, we still expected that increasing sample size would have overcome the potential variability in outcome.

This modest classification accuracy could reflect the poor classification accuracy of the single-center-illness-prediction models. It is possible that in small samples, differences between classes are not large enough to overcome sample variability (i.e. the signal-to-noise ratio was very low). Adding “low signal-to-noise centers” might have increased noise. Also, our multi-center models might have been more accurate with larger samples and if single-center models had a better performance. This would be consistent with what reported by Schnack et al. (2010), who examined the influence of noise and sample size in multicenter MRI studies.

### 4.4 Male-only multi-center illness course classification

To investigate whether reducing patient heterogeneity improved the classification models, we repeated the illness course classification analyses including only male patients. Combining all males into a multi-center-illness-prediction model led to an average accuracy of 54% overall. This was slightly better than the accuracy achieved by the model that included both sexes. The sample from São Paulo showed a significant improvement in accuracy of 12% of PPA and 17% of NPA compared to the multi-center mixed-sex models (p < 0.089 and p < 0.02 respectively). São Paulo's continuous group contained mainly male subjects, and could have benefited most by the exclusion of female subjects. While a decrease in sample size typically leads to a decrease in accuracy, this may have been compensated by an increase in signal-to-noise ratio in the more homogeneous sample (Schnack and Kahn, 2016). This increased homogeneity when considering only male patients could be due to a combination of factors. For example, there is evidence of sex-specific brain abnormalities associated with schizophrenia, and of sex-specific differences in illness characteristics, with males being likely to have an earlier age of onset, to experience more severe symptoms and to relapse more frequently (Aleman et al., 2003, Bryant et al., 1999, Goldstein, 2002, Leung and Chue, 2000). Single-sex models could therefore more easily find a “typical” predictive pattern in one sex group during the training process, which would be more difficult to obtain in the presence of sexual dimorphism.

### 4.5 Limitations

Together with the heterogeneity introduced by different neuroimaging acquisition parameters, the heterogeneity introduced by clinical factors may have played a major role in the accuracies we achieved. The instruments used to assess illness course differed across studies and this may have resulted in heterogeneous and overlapping patient groups. Furthermore, the length of follow up, although long for all groups, ranged from 3 to 7 years and one cannot exclude that illness course may become more established with time. Furthermore, some centers had more clinical information available (for example on duration of psychosis) which could have potentially allowed for more accurate decisions about illness course. This highlights the importance of gathering precise and standardized information on the duration and characteristics of each psychotic episode when classifying outcome, which could eventually result in better models.

Another limitation of this study is the size of the samples included in the analysis. Even though we had data from 5 centers, we decided to restrict our analyses only to those subjects with the two most extreme illness course types (remitting and continuous), which resulted in some centers having only a small sample size, with a particularly small number of female subjects. Finally, we only used one brain scan, and it is possible that a better prediction of illness course could be achieved by measuring change over time or studying trajectories of change (Cropley and Pantelis, 2014).

## 5 Conclusion

In summary, we provide proof of concept that combining multi-center MRI data to create a single, well performing model is possible. Theoretically, multi-center models could lead to more robust classification accuracy than models based on just a single center when using brain structure to predict illness course or diagnosis, which would be more generalizable to new patient samples. Multi-center models would consider scanner and acquisition protocols as noise and find effects common to all centers, reducing the risk of misclassification. However, the effect within each single center needs to be strong enough to contribute to the multi-center model. This effectively means that a center has to contribute a sample that is large or homogeneous enough to individually classify with significant accuracy, in order to be of use in generating robust multi-center models.

## Figures and Tables

**Table 1:** Demographic information on the samples.

	Institute of Psychiatry, Psychology and Neuroscience King's College London		University Medical Center Utrecht		University of Melbourne		University of Cantabria, Santander		University of São Paulo	
Clinical course (**R**emitting/**C**ontinuous)	**R**	**C**	**R**	**C**	**R**	**C**	**R**	**C**	**R**	**C**
Patients (males)	27 (13)	30 (22)	15 (14)	24 (21)	16 (9)	14 (12)	41 (21)	16 (12)	19 (9)	10 (8)
Age in years (SD)	28.1 (6.3)	29.2 (9.7)	22.2 (3.9)	24.6 (5.3)	21.3 (3.6)	21.6 (3.1)	31.4 (9.0)	29.8 (9.5)	26.2 (8.8)	31.7 (9.2)
DUP in days (SD)	245 (825)	579 (1052)	144 (190)	243 (425)	–	–	373 (577)	229 (281)	59 (82)	122 (199)
Schizophrenia diagnosis^a^	9	22	12	23	0	10	22	10	1	5
Sex (**M**ales/**F**emales)	**M**	**F**	**M**	**F**	**M**	**F**	**M**	**F**	**M**	**F**
Whole original sample^b^	61	36	58	9	37	17	62	45	38	26

a The number of patients diagnosed with schizophrenia at follow-up per sample per group.

b Sample including all patients with Remitting, Continuous and Intermediate course.

**Table 2:** Questionnaires used in each center.

	DSM-IV/DSM-V	ICD-10	PANSS	WHO life chart	SCAN	CASH	GAF	SAPS	SANS
London	x	x		x	x				
Utrecht	x		x			x	x		
Melbourne	x			x					
Santander	x	x						x	x
São Paulo	x		x				x		

**Table 3:** Scanner-protocols and scanner-type per center.

	Field strength	System	Sequence	Flip angle	Repetition time ms	Echo time (TE) ms	Voxel dimension (mm)^b^		
							x	y	z
University of Cantabria Santander	1.5 T	General Electric SIGNA System	SPGR^a^	45°	24	5	1.02	1.02	1.50
University Medical Center Utrecht	1.5 T	Philips	Fast field echo	30°	30	4.6	1.00	1.20	1.00
The University of Melbourne	1.5 T	General Electric SIGNA System	SPGR^a^	30°	14.3	3.3	0.94	0.94	1.50
Kings College London	1.5 T	General Electric SIGNA Systems	SPGR^a^	20°	13.8	2.8	0.94	1.50	0.94
University of São Paulo	1.5 T	General Electric SIGNA System	SPGR^a^	20°	21.7	5.2	0.86	0.86	1.50

a Spoiled gradient recalled acquisition in steady state.

b All the scans had a coronal acquisition orientation.

**Table 4:** Results of the classification models. The right hand side shows accuracies of the single-center models and the left hand side shows the accuracies of the multi-center models. Part (a) of the table shows the percentage of correctly classified males and females in the sex classification model; (b) shows the negative and positive predictive accuracies of the multi-center and single-center models on illness course classification; (c) shows the less heterogeneous illness course models, including only males.

(a) Gender classification, males vs. females						
			Multi-center model		Single center models	
	N males	N females	Male	Female	Male	Female
London	61	36	93%⁎	85%⁎	90%⁎	88%⁎
Santander	62	45	91%⁎	89%⁎	87%⁎	90%⁎
Utrecht	58	9	81%⁎	87%⁎	89%⁎	69%
Melbourne	37	17	94%⁎	89%⁎	87%⁎	78%⁎
São Paulo	38	26	87%⁎	86%⁎	89%⁎	79%⁎
Overall	256	133	90%⁎	87%⁎	88%⁎	81%⁎

(b) Illness course classification, continuous vs. remitting patients						

Entire sample			Multi-center model		Single center models	

	N continuous	N remitting	PPA	NPA	PPA	NPA

London	30	27	55%	55%	68%⁎	70%⁎
Santander	16	41	44%	45%	44%	42%
Utrecht	24	15	49%	48%	48%	48%
Melbourne	14	16	52%	51%	54%	53%
São Paulo	10	19	56%	62%	61%	62%
Overall	94	118	52%	52%	55%	55%

(c) Illness course classification, continuous vs. remitting patients						

Males only			Multi-center model		Single center models	

	N continuous	N remitting	PPA	NPA	PPA	NPA

London	22	13	62%	62%	64%	67%
Santander	12	21	46%	45%	53%	54%
Utrecht	21	14	45%	45%	47%	47%
Melbourne	12	9	55%	54%	53%	53%
São Paulo	8	9	68%	80%⁎	75%	78%
Overall	75	66	54%	54%	58%	60%

⁎ Significant models with p-value < 0.001.
